# Gastric Ulcer With Yttrium-90 Microsphere Selective Internal Radiation Therapy

**DOI:** 10.7759/cureus.58702

**Published:** 2024-04-21

**Authors:** Muhammad Mushtaq, Rawan Dayah, Brooke Corning

**Affiliations:** 1 Department of Internal Medicine, University of Texas Medical Branch, Galveston, USA

**Keywords:** y90-sirt, liver metastatic cancer, ablation therapy, upper gi bleeding, gastric ulcer

## Abstract

Radioembolization with yttrium-90 (Y90) is a recent oncological interventional radiology technique used to treat hepatocellular carcinoma and metastatic colon cancer to the liver. Although Y90 selective internal radiation therapy (Y90-SIRT) is considered a safe and effective treatment, with increasing use, hepatic and extrahepatic complications have been reported. Here, we present a case of upper gastrointestinal bleeding caused by gastric ulceration associated with radioembolization from Y90-SIRT, as confirmed by histological findings. Unlike dyspeptic ulcers, radioembolization ulcers originate on the serosal surface, predisposing patients to adhesions, bowel obstruction, or perforation, as well as gastrointestinal bleeding.

## Introduction

Radioembolization with yttrium-90 (Y90) is a recent oncological interventional radiology technique used to treat hepatocellular carcinoma (HCC) and metastatic colon cancer to the liver [1]. The method delivers localized high-dose radiation to tumors through the hepatic artery [2]. The therapy was first introduced in 1965, and with the development of microspheres that lodged further into the hepatic artery than parenchyma, it was approved for unresectable HCC in 1999 by the Food and Drug Administration (FDA) and shortly after for metastatic colon cancer to the liver [3]. Currently, Y90 is used for tumor control as a bridge to transplant or resection, in the downstaging of tumors for curative treatment, as well as a curative therapy when used for segmentectomy [3]. Due to the increased utilization of the therapy, hepatic and extrahepatic complications have been reported.

## Case presentation

Our patient was a 59-year-old man with a past medical history of stage IV adenocarcinoma of the rectum (with bilobar hepatic metastasis and nodal involvement). Other medical comorbidities include hypertension, hypertriglyceridemia, prediabetes, and gout. He received FOLFIRI (folinic acid, 5-fluorouracil, and irinotecan) and Avastin, but unfortunately, he had a progression of liver lesions and underwent two treatment sessions of Y90 selective internal radiation therapy (SIRT). Shortly after his second Y90-SIRT, he developed coffee ground emesis and black stool. On presentation to the emergency department, vitals included a pulse of 115 and blood pressure of 113/79. Significant labs included a hemoglobin of 16.5 g/dl, mean corpuscular volume (MCV) of 100.4 fL, and blood urea nitrogen (BUN) of 20 mg/dl. A CT of the abdomen with IV contrast showed thickening of the gastric antrum and duodenal bulb along with nodular thickening in the posterior wall of the mid-rectum (measuring 3.1 cm thickness and spanning about 7.9 cm), with perirectal fat stranding, concerning for primary neoplastic process and fluid density low attenuating lesions seen at the liver concerning for metastatic lesions. 

**Figure 1:**
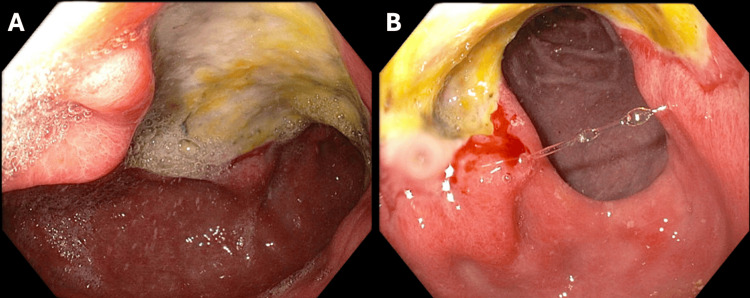
EGD on initial presentation demonstrating cratered ulcer with a flat pigmented spot (Forrest classification IIc) at the pylorus extending into the gastric antrum (A), along the incisura and the duodenal bulb (B), 40 mm in largest dimension. EGD: esophagogastroduodenoscopy

Esophagogastroduodenoscopy (EGD) was performed, showing a cratered ulcer with a flat pigmented spot (Forrest classification IIc) at the pylorus extending into the gastric antrum, along the incisura and the duodenal bulb, 40 mm in largest dimension (Figure 1).

**Figure 2:**
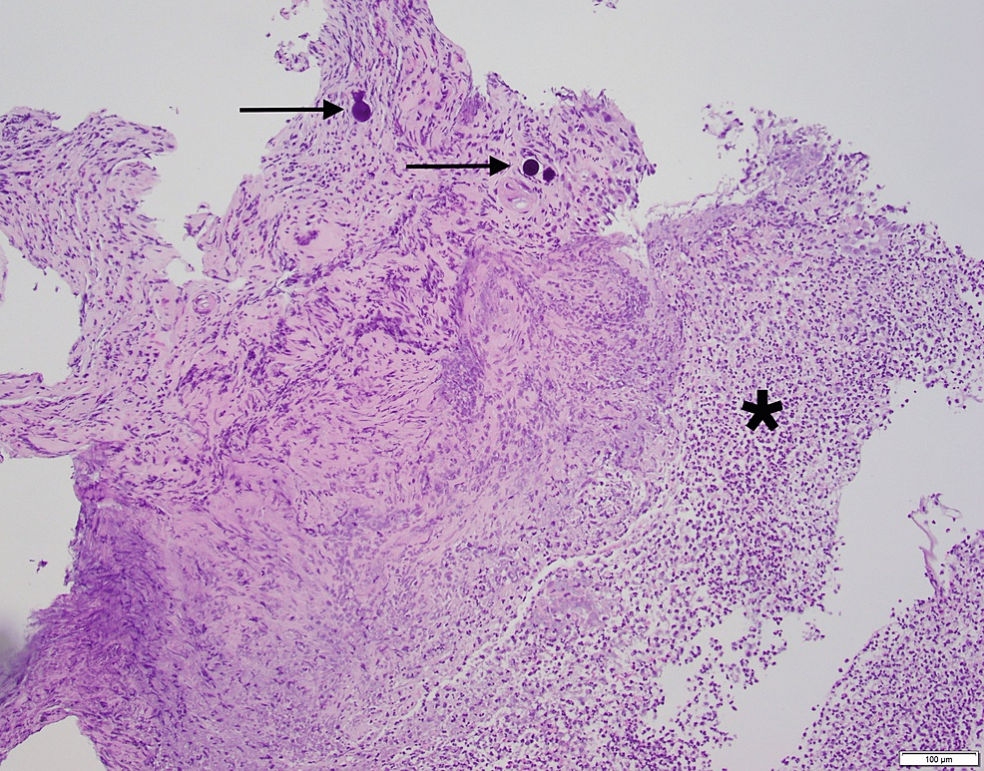
Histopathology showing reactive or regenerative changes of the gastric mucosa with intravascular or intracapillary Y90 particles. Y90: yttrium-90; right arrow: Y90 microspheres; *: tissue regenerative changes

A biopsy was performed, which showed features of ischemic necrosis, inflammatory exudate, and regenerative changes, along with evidence of Y90 microspheres (Figure 2). The patient remained stable with the resolution of bleeding and was discharged home on proton pump inhibitor therapy. A repeat EGD eight weeks later showed improvement in the ulcer, with size reduced to 30 mm (Forrest classification III) with complete resolution of symptoms. Given the gastric ulcer finding, further treatment with Y90 was discontinued, and the patient was transitioned to treatment with FOLFOX and bevacizumab. 

## Discussion

The standard of treatment for liver lesions either secondary to HCC or metastatic disease from colon cancer is surgical resection combined with adjuvant chemotherapy or liver transplant. However, depending on the location of the lesion, proximity to the vasculature, size of the lesion (>3 cm), and the patient's overall surgical risk, the surgical approach can be controversial. In those cases, locoregional interventions are considered, including ablation and radioembolization. Ablation becomes limited if the lesion is close to the vasculature. In instances where ablation is contraindicated, transarterial chemoembolization (TACE) is considered the first-line radioembolization technique. The limitations of TACE include the presence of portal vein thrombosis (PVT) since it can lead to acute liver failure. Thus, Y90-SIRT was initially introduced as a locoregional embolization therapy in the context of PVT, where TACE was contraindicated. However, subsequent studies have shown it to be as effective as TACE in patients with or without PVT [3]. Furthermore, due to its minimal embolic nature, Y90 treatment does not prevent other arterial therapies in the future, i.e., TACE and hepatic artery infusion chemotherapy [3].

Radioembolization with Y90 involves the delivery of Y90-infused glass microspheres under fluoroscopic guidance through the femoral artery into the hepatic arterial blood flow, delivering therapy to the tumor. The tumor receives a highly concerted radiation dose to spare healthy liver parenchyma due to the preferential blood supply of non-tumor parenchyma from portal venous blood [4]. With increased utilization of the therapy, hepatic and extrahepatic complications are reported, including radioembolization-induced liver disease (REILD, 20%), gastrointestinal complications (radiation cholecystitis, acute pancreatitis, cholangitis, and gastrointestinal ulcers) (0-13%), radiation pneumonitis (<1%), radiation dermatitis (rare), and lymphopenia (rare) [5-8].

Gastric ulcers have been reported in less than 5% of the patients undergoing Y90-SIRT [6]. Reflux of Y90-loaded spheres into the gastroduodenal artery has been reported to cause gastroduodenal ulceration. It is recommended to embolize the gastroduodenal artery with metal or hydrofoils to avoid reflux [5,6]. Gastric ulceration cases have also been reported due to vasculature variation and retrograde migration of Y90 microspheres into the gastric or duodenal circulation [8]. Patients usually present with abdominal pain, nausea, vomiting, or hematochezia. Endoscopy is required to differentiate from other radioembolization-associated complications. EGD findings include erythema, erosion, edema, or ulceration [7,9]. A definitive diagnosis is made by endoscopic biopsy, showing the radioembolic microspheres within the capillaries, which was evident in our patient's case (Figure 2). Unlike dyspeptic ulcers (which are mucosal defects), radioembolization ulcers originate on the serosal surface of the viscera, which can predispose patients to adhesions, bowel obstruction, perforation, and gastrointestinal bleeding [9]. The patients with Y90-induced gastric ulcers are managed with proton pump inhibitors effectively [6], as was the case in our patient.

## Conclusions

After Y90-SIRT treatment, clinicians must remain vigilant for the potential development of gastric ulcers in patients experiencing gastrointestinal symptoms within a few months of therapy. Unlike dyspeptic ulcers, radioembolization ulcers initiate on the serosal surface, which may lead to adhesions, bowel obstruction, perforation, and gastrointestinal bleeding. Therefore, it is imperative to promptly identify symptoms and provide the necessary intervention to prevent negative consequences. Patients undergoing Y90-SIRT may also benefit from prophylactic proton pump inhibitor therapy, offering a promising area for future research.
